# Quantitative Structure-Activity Relationship, Ontology-Based Model of the Antioxidant and Cell Protective Activity of Peat Humic Acids

**DOI:** 10.3390/polym14163293

**Published:** 2022-08-12

**Authors:** Maria V. Zykova, Konstantin S. Brazovskii, Kristina A. Bratishko, Evgeny E. Buyko, Lyudmila A. Logvinova, Sergey V. Romanenko, Andrey I. Konstantinov, Sergei V. Krivoshchekov, Irina V. Perminova, Mikhail V. Belousov

**Affiliations:** 1 Pharmaceutical Faculty, Siberian State Medical University, Tomsk 634050, Russia; 2 Research School of Chemistry & Applied Biomedical Sciences, National Research Tomsk Polytechnic University, Tomsk 634050, Russia; 3 Department of Chemistry, Lomonosov Moscow State University, Leninskiye Gory 1-3, Moscow 119991, Russia

**Keywords:** humic acids, antioxidant activity, ontology model, QSAR

## Abstract

Peat humic acids are well known for their wide range of biological effects which can be attributed to the complex chemical structure of naturally occurring humic substances. One of the promising tools is an ontology-based quantitative analysis of the relationship between physical and chemical parameters describing a chemical structure of peat humic acids and their biological activity. This article demonstrates the feasibility of such an approach to estimate the antioxidant and cell protective properties of the peat humic acids. The structural parameters of the peat humic acids were studied by electronic, fluorescence, infrared, ^13^C-NMR spectroscopy, titrimetric analysis, elemental C,H,N, and O- analysis, and gel chromatography. Antioxidant and antiradical activities were assessed by physicochemical methods of analysis: electronic paramagnetic resonance, cathodic voltammetry, ABTS^•+^ scavenging, assay of DPPH radical-scavenging activity, assay of superoxide radical-scavenging activity, iron chelating activity, and scavenging of hydroxyl radicals. Cytoprotective activity was evaluated by the neutral red-based cytotoxicity test in 3T3-L1 cell culture in a wide range of concentrations. Assessment of intracellular ROS production was carried out using a 2,7-dichlorodihydrofluoresceindiacetate (DCFDA) fluorescent probe. Intracellular ROS production was induced using two common prooxidants (tert-butyl hydroperoxide, Fe^2+^ ions). We suggested an ontology-based model for the antioxidant and cytoprotective activity of humic acids based on experimental data and numerical models. This model establishes the way to further research on the biological effects of humic acids and provides a useful tool for numerical simulation of these effects. Remarkable antioxidant and cell protective activity of humic acids makes them a promising natural source of new pharmaceutical substances that feature a wide range of biological effects.

## 1. Introduction

Free radicals are unstable molecules containing at least one unpaired electron in the external orbital [1]. However, in the 1950s it was discovered that free radicals play a significant role in many pathological and aging processes [2]. The redox balance is the key factor of all processes occurring in both normal and pathological cells because reactive oxygen species (ROS) could imitate some molecular messengers and severely disturb normal metabolic pathways. The broken oxidation-reduction equilibrium caused by either the excessive production of ROSs or inadequate activity of the endogenous antioxidant protective system inevitably results in the oxidative stress [3].

There are some exogenous naturally occurring molecules, mainly nutrients, possessing the antioxidant activity. They include carotenoids, vitamins E and K, ascorbic acid, flavonoids and phenol carbonic acids, zinc, selenium and many other chemicals. Humic acids (HAs) also could be considered as the effective antioxidants; their high potential to reduce active free radicals has been reported by many research groups [4,5,6,7,8,9,10,11,12,13,14,15,16,17]. According to the commonly accepted opinion, HAs are naturally occurring high- and super-molecular polyfunctional colloidal bioorganic molecules featuring both amphoteric and amphipathic properties. The antioxidant activity of HAs could be attributed to the abundance of phenolic hydroxyl, quinoid and other chemical groups having a highly delocalized molecular orbital. As a result, HAs can donate protons, catch free radicals and chelate reactive ions under normal physiological conditions. Moreover, being potent antioxidant and free radical catcher substances, HAs do not display any specific toxic activity toward cells, tissues, or organisms [18,19,20,21]. Owing to this fact, humic substances are a promising natural source of new raw materials for food and pharmaceutical industries [12,22,23]. Though very promising as a potential new drug the chemical structure of HAs has not yet been identified and the quantitative structure-activity relationship (QSAR) remains unmodeled. The main reason for that is the exacerbated chemical complexity of HAs. One more important factor is that the samples of HAs obtained from different sources have quite a remarkable variation in their physical and chemical properties and are accompanied by nonlinear relationships between these parameters and biological activities. As a result, typical statistical models, such as a multiple linear regression, are not relevant enough to produce a practically significant prognosis. 

This fact severely limits the possibility of focused research and creation of new potent antioxidants and cell protectors based on HAs. These obstacles, at least in theory, could be overcome through application of artificial neural networks (ANNs) due to their potential to approximate a very wide range of functions, including nonlinear QSARs of a moderate complexity. With some reasonable precautions, ANNs could be considered as a powerful framework to model complex biopharmaceutical systems [24,25]. ANNs successfully predicted antioxidant activity of essential oils [26], beetroot extracts [27], teas [28], and phenolic compounds extracted from strawberries [29]. In addition, neural networks effectively complement common analytic methods, enhancing data processing protocols when it comes to characterization of very complex biochemical systems [30].

Recently proposed state-of-the-art machine learning techniques, for example, the self-organizing feature map (SOFM), made it possible to find the collective and individual characteristics of HAs obtained from different geographic locations [31]. One more application of ANNs is to simulate physical and chemical properties of supramolecular structures. A backprojection neural network trained on experimental UV absorption spectra demonstrated high accuracy predicting optical parameters during coagulation of HAs [32]. Finally, artificial neural networks are among the best tools to optimize parameters of chemical processes that consider humic acids [33]. Unfortunately, ANNs have several inherent drawbacks that significantly restrain their use as an analytical tool [34]. Primarily, ANNs tend to over-fit experimental data, which reduces the overall accuracy of the model. One more unwanted phenomenon is lack of transparency and ambiguous structure of the artificial networks. As a result, ANN-based QSAR models in many cases cannot be interpreted in physical, chemical and biological terms. These disadvantages become even more important when one studies relationships between the structure of complex macromolecules and their biological activities.

Recently we reported our results on a new approach to modeling immunotropic effects of humic acids extracted from peat [35]. The proposed ANN model uses spectral parameters of the samples in visible and near-ultraviolet range to estimate the production of nitric oxide by activated peritoneal macrophages. This approach is useful for screening and preliminary selection of the samples because it requires only basic measurements of the absorbance spectrum.

The limitations of ANN-based QSAR models are becoming increasingly more evident as the complexity of the structure-activity relationships increases. There are three major drawbacks that must be overcome to build an adequate ANN model of a complex system with sufficient non-linear dependencies between inputs and output. First, the input variables have to be carefully selected to provide distinguishability of the samples. Second, both training and testing datasets must be representative, meaning their size and variation of values. Last, but not least, the structure of the artificial neural network must be defined before any training and testing runs. In the case of complex multistage processes, for example, antioxidant activity, ANN models could produce practically meaningless results. We believe that this is the main reason why this approach is not the mainstream modeling tool used to describe complex biological processes.

Counterintuitive enough, but simpler methods, for instance, multiple regression occasionally work better and produce meaningful interpretable results. The first systematic study of antioxidant activity of humic acids having different origin and composition was reported in [7]. The major components of humic acids with the highest antioxidant activity have been identified and described as a linear multiple regression model with only four independent variables: atomic C/N ratio, content of O-substituted methine and metoxy groups and total content of phenols.

One more successful example of the regression-based QSAR model was published in [36]. The authors built a regression model that involves NMR parameters of humic-like substances extracted from lignin in agro-industrial byproducts. Similar results were reported in [37], where a principal components model was implemented. This type of model is also relatively simple and features ability to unveil the structure of the underlying parameters. Research has also been performed on potential antiviral activity of humic substances; for example, [38] reported dependency of the antiviral activity on parameters measured with NMR and mass spectrometry.

Summarizing the published results, it can be concluded that although artificial neural networks are a very powerful tool to model a quantitative structure-activity relationship, they have inherent disadvantages limiting their ability to describe complex multistage biological processes. The simpler models based on a multiple regression or linear vector decomposition occasionally produce more robust and self-explainable predictions.

This paper focuses on new approach to model antioxidant and cell protective activity of humic acids extracted from peat. We propose the so-called ontology-based model that allows integration of heterogeneous models into a single framework [39]. The ontology-based modeling is a reliable and effective approach that finds application in many research areas, for example, the Gene Ontology Consortium (http://geneontology.org, accessed on 30 May 2022). There have been no publications found on building QSAR models using ontology-based principles and methods. Here we introduce this approach to model antioxidant and cell protective activity of humic acids extracted from peat.

## 2. Materials and Methods

### 2.1. Materials

Humic acids were extracted from nine samples of minerotrophic, mesotrophic and oligotrophic peat types, using basic (NaOH) and pyrophosphate (Na_4_P_2_O_7_) extractions were designated as HA_alk_ and HA_pyr_, respectively.

The nine representative types of peat [40] were taken from major peat bogs in the Tomsk region, Russia: the oligotrophic bog located in the southern taiga zone between the Iksa and the Bakchar rivers, representing the north-eastern spurs of the Great Vasyugan Mire (56°58′ N latitude and 82°36′ W longitude; peat samples 1–4, 6, 7, and 9), and the eutrophic bogs of Klyukvennoe (56°23′ N latitude and 84°42′ W longitude; peat sample 5) and Tagan (56°21′ N latitude and 84°48′ W longitude; peat sample 8). Different peat profiles in the bogs were chosen, and samples were taking from different depths of the peat sections (Table 1).

The degree of decomposition of the peat samples was estimated microscopically (at magnification ×56–140) as a ratio of groundmass to volumetric amount of tissues contained in the peat samples. The HAs extracts were prepared following the standard procedure. Firstly, the peat samples were desiccated at room temperature, milled, and incubated with 0.1 M Na_4_P_2_O_7_ (pyrophosphate extraction) or 0.1 M NaOH (basic extraction) for 5–8 h at 30–50 °C under continuous mixing. Then, the precipitate was filtered out and the remaining solution was treated with HCl at pH 1.0–2.0.

Finally, the mixture was centrifuged, washed on the filter with water to increase pH up to 7.0, and dried at room temperature. Yield of HAs from the peat samples was measured by the gravimetric method. The yield of the alkaline extraction is 1.5–3 times higher in comparison to the pyrophosphate (Na_4_P_2_O_7_)-based method. The samples of peat-derived HAs are amorphous dark brown odorless powder.

### 2.2. Characterization of Humic Acids

The optical properties of HAs were studied with visual light spectroscopy of the aqueous solutions (0.001% mass) in quartz cuvettes (1 cm) using an Unico 2800 spectrophotometer (UNICO, Dayton, NJ, USA). The optical absorbance at 465 nm (A_465_) and 650 nm (A_650_) were measured, then the A_465_/A_650_ ratio was calculated (Table S1).

**Figure 1:**
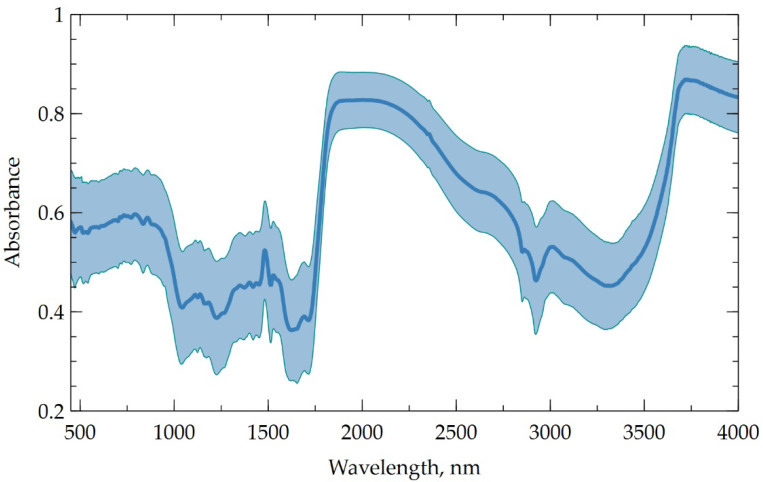
Averaged IR spectrum of the peat samples.

The infrared (IR) spectra of HAs mixed with KBr in the proportion 1:100 were registered in the IR region from 500 to 4000 cm^−1^ with IR-Fourier spectrometer FSM 2201 (Infraspek Ltd., Saint-Petersburg, Russia). The IR spectrum does not feature specific peaks and is highly variable among the samples. The averaged spectrum is given in Figure 1; Table S2 contains detailed IR-related parameters for each sample. The spectral parameters were selected following the method described in [41]; relative quantity of HAs functional groups in the peat samples was estimated through optical absorption in spectral ranges associated with oxygen-containing hydroxyl groups (υ_OH_, 3400 cm^−1^), carbonyl groups (υ_C=O_, 1720 cm^−1^), ester (υ_C-O, C-O-C_, 1225 cm^−1^), and alkyloxy groups (υ_C-O_, 1035 cm^−1^), divided by optical absorption of aromatic (υ_C=C_, 1610 cm^−1^) and aliphatic (υ_Aliphatic_, 2920 cm^−1^) fragments of the HA molecular structure.

To measure total acidity, the HAs samples were treated with a Ba(OH)_2_ solution under N_2_ atmosphere for 24 h. The Ba(OH)_2_ remaining in the solution after the reaction was then back-titrated with a standard acid solution. For the titration of carboxylic acid groups, the HA samples were treated for 24 h with calcium acetate solution in excess, which causes the release of acetic acid. The CH_3_COOH released was then titrated with a standard base solution. Phenolic OH groups were calculated as the difference between total acidity and acidity of the carboxylic groups (Table S1).

The elemental (C, H, N, O, S) composition of HAs was determined by combustion using CHNS Flash 2000 (Thermo Fisher Scientific, Cambridge, UK); the O_2_ content was calculated as the difference (Table S1).

The quantitative solution-state ^13^C-NMR spectrum of HAs was recorded with a Bruker NMR spectrometer AVANCE 400 (400 MHz, Bruker, Billerica, MA, USA) operating at 100 MHz; the mode was INVGATE; the pulse sequence was CPMG; the first sequence pulse was 90°; the registration time of the free induction decay signal was 0.2 s; the relaxation delay time was 7.8 s, and the recording duration of one ^13^C–NMR spectrum was about 12 h [42]. The spectra obtained were quantified and the assignments were made (in ppm): 0–48 aliphatic H and C substituted C atoms (C_CHn_); 48–59 methoxyl C atoms (C_CH3O_); 59–66 O-substituted methylene groups (C_CH2O_); 66–91 O-substituted methine groups (C_CHO_); 91–108 anomeric double substituted aliphatic C atoms (C_OCO_); 108–145 aromatic H- and C-substituted atoms (C_Ar_); 145–168 aromatic O-substituted C atoms (C_Ar-O_); 168–189 C atoms of carboxylic and esteric groups (C_COO_); and 189–220 C atoms of quinone and ketone groups (C_C=O_). In addition to the integrals of the given ranges, the sum of O-substituted aliphatic C ΣC_Alk-O_, carbohydrate carbon ΣC_Carb_ and the ratio ΣC_Ar_/ΣC_Alk_ was calculated. The value of ΣC_Alk-O_ was a sum of C_OCHO_+C_CHO_+C_CH2O_+C_CH3O_; ΣC_Carb_ was a sum of C_OCO_, C_CHO_, and C_CH2O_; and ΣC_Ar_ and ΣC_Alk_ were the sums C_Ar_+C_Ar–O_ and ΣC_Alk-O_+C_CHn_, respectively (Table S2).

The molecular weight distribution of HAs was determined by HP-SEC using an Ultrahydrogel 250 column (300 × 7.8 mm, 6 µm, pore size 250 Å; Waters, Milford, MA, USA). The mobile phase was 0.1 M Tris-HCl, pH 8.9 (1 mL/min). A Dionex Ultimate 3000 chromatograph (Thermo Fisher Scientific/Dionex, Waltham, MA, USA) with a vacuum degasser was used, LPG-3400SD pump, column thermostat TCC-3000SD, and a spectrophotometric detector DAD-3000 operating at 240 nm. The molecular weights of the fractions were estimated by comparison with the retention times of polystyrene sulfonate standards (PSS Polymer Standards Service GmbH, Mainz, Germany) (Table S1).

The excitation-emission fluorescent (EMM) spectrum of the samples was recorded with a PerkinElmer 6500 fluorescence spectrometer using standard 10 mm cuvettes for fluorescence measurements. The emission spectra were in range from 200 to 500 nm, step 1 nm. The excitation spectra were from 200 to 475 nm, step 25 nm (Figure S1). 

All spectra were processed using the staRdom package (https://github.com/MatthiasPucher/staRdom, accessed on 30 May 2022) in the statistical calculation environment Rstudio (version 2021.09.0) statistical calculation environment (https://www.rstudio.com, accessed on 30 May 2022). As a preprocessing step, first and second order Rayleigh and first order Raman scatter were removed, after which the spectra were normalized.

### 2.3. Free Radical Scavenging Activity of Humic Acids

The antioxidant activity of HAs was determined using assay of 2,2-diphenyl-1-picryl-hydrazyl-hydrate (DPPH) radical-scavenging capacity test. The bleaching reaction was registered with a Unico 2800 (UNICO, Dayton, NJ, USA) spectrophotometer at 520 nm. The scavenging activity was expressed as the percent of the inhibited radicals (Table S3).

Additionally, the antioxidant potential was measured as the electrochemical reduction current of O_2_ at a mercury film electrode after reaction of the compounds with O_2_^−^ in phosphate buffer solution with pH = 6.9 [43]. The HAs were added to the electrochemical cell at concentration of 1 µg/mL and stirred. Voltammograms of O_2_ cathodic reduction were recorded using linear sweep voltammetry with potential scan rate 30 mV/s and potential range from E = 0 to −0.7 V. The electrochemical cell included a working mercury film electrode and a silver/silver chloride reference electrode in saturated KCl (Ag|AgCl|KClsat) solution; HAs did not adhere to the surface of the working mercury film electrode in the range of O_2_ reduction [43]. Test compounds reacted with ROS and changed the electrochemical reduction current of O_2_ (first wave at E = −0.3 V). The voltammograms were used to plot time dependences of the function [1 − *I*/*I*_0_] in the presence of a test sample. The linear part of the plot and the slope ratio of the tangent to this portion of the curve were used to calculate the kinetic criterion of antioxidant activity, K (μmol/L × min^−1^) (Table S3).

Total antioxidant capacity of HAs was evaluated using ABTS assays and a Unico 2800 (USA) spectrophotometer. Absorption was measured at 734 nm. HAs interact with a stable free radical cation ABTS (diammonium salt of 2,2′-azino-di-(3-ethylbenzthiazolinsulfonic acid) reducing its content in the reaction mixture [44]. The radical scavenging activity was expressed as the IC_50_—the concentration of HAs, at which the concentration of ABTS decreased by 2 times. “Trolox” (Acros Organics, Bratislava, Slovakia) was used as a positive control (Table S3).

The concentration of semiquinone-type free radicals of HAs was estimated through the number of paramagnetic centers (PMC) by EPR spectroscopy using a Bruker EMX EPR spectrometer (EPR Division, Bruker Instruments, Inc., Billerica, MA, USA) at room temperature (~23 °C). The absolute concentration of the unpaired spins was calculated against a standard CuCl_2_·5H_2_O solution containing a known number of PMC (Table S3).

The interaction of HAs with the superoxide-anion radical (O_2_^−^) was studied through measuring the concentration of O_2_^−^. To maintain stable concentration of O_2_^−^·a non−enzymatic O_2_^−^·generation system was used [45], where electrons with NADH+H+ are transferred via phenazine metasulfate (FMS) to molecular oxygen forming superoxide that reduces nitro blue tetrazolium (NST) into formazan [46]. A Unico 2800 (USA) spectrophotometer was used to measure absorption at 560 nm. The radical scavenging activity was expressed as the indicator IC_50_–the concentration of HAs, at which the concentration of NST decreased by 2 times. Ascorbic acid (Sigma Aldrich, Merck Life Science LLC, Moscow, Russia) was a positive control (Table S3).

The specific iron chelating activity was estimated through reaction between HAs and ferrosine-Fe^2+^ complex [46] detecting optical absorption with a Unico 2800 (USA) spectrophotometer at 562 nm. This method evaluates the ability of HAs to bind Fe^2+^ ions, thereby suppressing lipid peroxidation (POL) and a lipid oxidation process caused by free radicals. Ferrozine (monosodium salt of 3-(2-pyridyl)-5,6-diphenyl-1,2,4-triazine-p,p′-disulfonic acid) forms a purple complex with Fe^2+^ ions. The iron chelating activity was expressed as the indicator IC_50_—the concentration of HAs, at which the ferrozine-Fe^2+^ complex in a model system decreased by 2 times. Ethylenediaminetetraacetic acid (EDTA) (Sigma Aldrich, Merck Life Science LLC, Moscow, Russia) was a positive control (Table S3).

Hydroxyl radical (HO·) is a very powerful oxidizing agent that reacts with almost all biomolecules found in living cells. HO generation was carried out during the Haber–Weiss reaction in the presence of deoxyribose. During this reaction deoxyribose was degraded to malondialdehyde (MDA). The latter was determined by the reaction with thiobarbituric acid (TBA), which at high temperature and acidic pH proceeds with the formation of a colored trimethine complex with an absorption maximum at a wavelength of 532 nm [46]. Owing to the high chelating activity of HA samples, it is necessary to take into account the possibility of Fe^3+^ binding by HA molecules, which can lead to a decrease in the concentration of the hydroxyl radical. It is known that EDTA binds Fe^3+^ ions into a complex that is capable of generating HO [46], so the ability of HA to bind HO• was studied in a model system with and without EDTA. The samples contained 20 mM KH_2_PO_4_-KOH buffer (pH 7.4), 0.1 mM ascorbic acid, 2.8 mM deoxyribose, 1 mM H_2_O_2_ and 0.1 mM FeCl_3_ or 0.1 mM FeCl_3_ and 1 mM EDTA, pre-mixed in equal volumes. In the experimental samples solutions HAs samples were added at final concentrations: 0.5, 1, 1.5, and 2 mg/mL. The control and experimental samples were incubated for 1 h at 37 °C, after which 1 mL of 0.5% TBA and 1 mL of 10% TCA (Trichloroacetic acid) were added to 0.5 mL of the reaction medium, placed in a boiling water bath for 15 min, cooled, and centrifuged. The optical density of the supernatant was measured using an SF2000 spectrophotometer (OKB Spectr LLC, Saint-Petersburg, Russia) at a wavelength of 532 nm. Based on the dose–response curve, the concentration of the HA sample at which 50% inhibition of MDA formation from deoxyribose was calculated. Mannitol (Thermo Scientific Acros, Loughborough, UK), a classical trap of hydroxyl radicals, was used as a standard (Table S1).

### 2.4. Cytotoxicity Study

The 3T3-L1 fibroblasts cell line was obtained from the State Scientific Center of Virology and Biotechnology “Vector”, Novosibirsk, Russia. The effect of HAs on the viability of 3T3-L1 normal fibroblast cell line was assessed using a neutral red test as described [47].

The 3T3-L1 cells cultivation was under standard conditions (5% CO_2_ atmosphere, DMEM/F-12 medium (Gibco^TM^, Billings, MT, USA) + 10% FBS (Gibco^TM^, Billings, MT, USA) + 2 mM L-glutamine (Gibco^TM^, Billings, MT, USA) and 1% gentamicin (Gibco^TM^, Billings, MT, USA). Aqueous solutions of the studied HAs samples were added in the concentration range 5.5–700 µg/mL. Cell plates were placed into a CO_2_ incubator for 24 h. After washing cells with 1 × PBS, 40 μg/mL neutral red working solution was added into wells for 2 h at 37 °C. To extract the dye, 150 μl mixture of 96% ethanol: deionized water: glacial acetic acid (50:49:1) was used. Optical density was measured at 540 nm and a reference wavelength of 650 nm using a multifunctional microplate reader Tecan Infinite 200 pro mplex (Tecan Group Ltd, Männedorf, Switzerland).

### 2.5. Intracellular Humic Acids Distribution Assay

The assay was conducted as described with minor modifications [48]. 3T3-L1 fibroblasts cells were cultured in complete medium (DMEM/F-12 (Gibco^TM^, Billings, MT, USA) +10% FBS (Gibco^TM^, Billings, MT, USA) + 2 mM L-glutamine (Gibco^TM^, Billings, MT, USA) and 1% gentamicin (Gibco^TM^, Billings, MT, USA) for at least 3 passages. After seeding the 24 well plate (0.05 × 10^6^ cells per well) and achieving 70–80% confluence, aqueous solutions of the HA samples were added at a final concentration of 25 μg/mL. After 24 h of incubation, cells were transferred to separate Eppendorfs, pelleted by centrifugation (2000 rpm, 5 min), and smeared on Cytoslide coated slides using a Thermo Scientific Cytospin 4 cytocentrifuge (Thermo Scientific, Waltham, MA, USA) (800 rpm, 3 min). Cells were fixated by 4% paraformaldehyde solution, permeabilized with 0.01% Triton solution, and covered with DAPI mounting media. Fluorimetric detection of intracellular HA distribution was carried out using a Leica DMi8 fluorescence microscope (Leica Microsystems, Wetzlar, Germany) by intrinsic fluorescence of HAs at λ_ex_ = 532–558 nm, and λ_em_ = 570–640 nm. Cell nuclei were visualized using the DAPI channel (λ_ex_ = 325–375 nm, and λ_em_ = 435–385 nm).

### 2.6. The Effect of Humic Acidss on the Action of Prooxidants In Vitro

Assessment of intracellular ROS production was carried out using a 2,7-dichlorodihydrofluoresceindiacetate (DCFDA) fluorescent probe. Intracellular ROS production was induced using two common prooxidants (tert-butyl hydroperoxide (t-BHP) and Fe^2+^ ions (FeSO_4_)).

The 3T3-L1 cells were cultured under standard conditions (5% CO_2_ atmosphere, DMEM/F-12 medium (Gibco^TM^, Billings, MT, USA) + 10% FBS (Gibco^TM^, Billings, MT, USA) + 2 mM L-glutamine (Gibco^TM^, Billings, MT, USA) and 1% gentamicin (Gibco^TM^, Billings, MT, USA)) and seeded on black 96-well culture plates for fluorescence measuring (1 × 10^4^ cells/well). The HAs (12.5 μM) or Trolox (10 μM) were added to the corresponding wells and incubated for 24 h. Then the cells were washed from the samples, and a working solution of DCFDA (10 μM) (Sigma-Aldrich, USA, D6883) was added to the wells. The plates were incubated in a thermostat for 20 min at 37 °C, washed from DCFDA, and stimulated with prooxidants 25 μM t-BHP or 10 μM FeSO_4_. After incubation for 60 min at 37 °C, the fluorescence in the wells was determined at λ_ex_ = 485 nm and λ_em_ = 530 nm using a multifunctional microplate reader, Tecan Infinite200 pro mplex (Switzerland).

## 3. Results

### 3.1. Antioxidant Activity of the Samples

The overall antioxidant activity of the studied samples corresponds to the published results within the reasonable range of uncertainty. Owing to high variability of the measured parameters, their central tendencies (the mean, median, and mode) provide essentially no useful information on the common properties of the samples. Instead, we describe our dataset using minimum and maximum values measured in experiments. Table 2 summarizes parameters of the antioxidant activity.

Obviously, antioxidant activity of the peat samples varies far beyond the expected natural fluctuations. The large uncertainty of these parameters is a well-known fact. Although there have been many hypotheses, a reliable antioxidant activity QSAR model has not yet been formulated. The commonly agreed opinion is that antioxidant activity of HAs can be attributed to phenolic compounds of different chemical structures. This hypothesis was checked by building linear regression models that include parameters of antioxidant activity and concentration of hydroxyl and carboxyl groups. The results are given in Table 3 and Table 4.

Unfortunately, the simplest linear model cannot support theory that antioxidant activity depends on concentration of hydroxyl and carboxyl groups. The main reason is that relationship between these parameters are much more complex and sufficiently nonlinear. One more explanation formulated in framework of ontology could be that OH and COOH groups are not the elementary, basic components of free radical scavenging processes.

Table 5 represents linear model relating the number of paramagnetic centers (NPC) and antioxidant activity of HAs. There is only one significant linear correlation between DPPH and NPC, but this model could explain only 30% of total variance. For brevity, we omitted the full linear regression analysis of the dataset because this model is not relevant to the process being studied.

Thus, the QSAR model of antioxidant activity of HAs cannot be described with simple or multiple linear regression. More complex models, for example, based on parallel factor analysis (PARAFAC) [37], reportedly demonstrate better prognostic quality and more reproducible results. 

**Figure 2:**
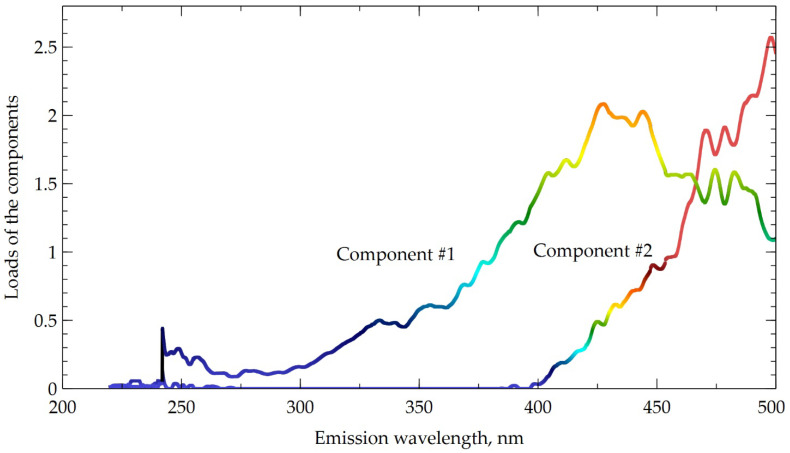
Components of PARAFAC model and their loads.

The PARAFAC model linking the EMM spectrum of the samples and their antioxidant activity contains two components (Figure 2). The concordance of the model was 98% that means good approximation of the EMM spectrum with a quite simple two-component model.

The PARAFAC model also demonstrates good prognostic properties towards parameters of antioxidant activity (Table 6). The components of the PARAFAC model can be assigned to phenolic compounds [37] in complete agreement with the accepted theory.

### 3.2. Cell Protective Activity of the Samples

**Figure 3:**
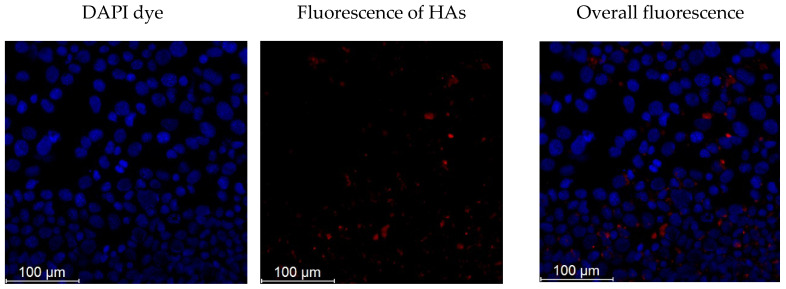
Fluorescence of the incubated cells in presence of humic acids.

The main cell protection system includes several intracellular pathways that reduce reactive oxygen species (ROS). The induced oxidative stress test proved that cell protective activity of the HAs is closely related to their antioxidant activity. Moreover, HAs can pass through the cellular membrane and interact with intracellular anti-ROS pathways. Figure 3 shows microphotographs of the sample taken with a fluorescent microscope.

**Figure 4:**
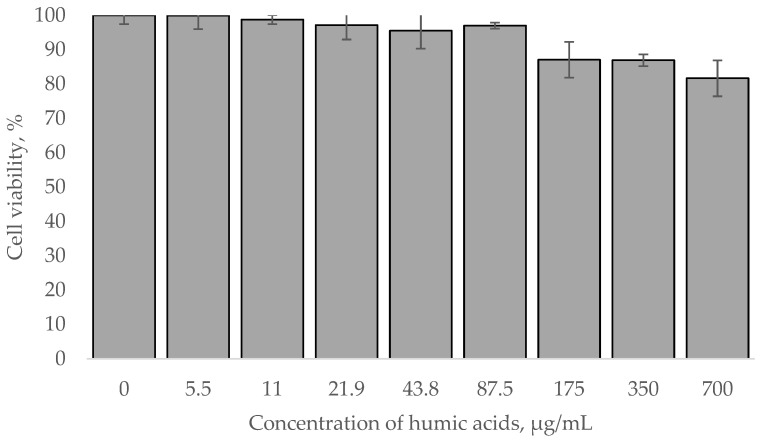
Viability of 3T3-L1 normal fibroblast cells incubated with HAs.

The fluorescent cells were incubated in presence of HAs in concentration up to 25 μg/mL, whereas control cells did not emit a detectable fluorescent signal. These concentrations of HAs could be toxic toward cells, but our experiments confirmed that even higher concentrations demonstrated weak cytotoxic effect (Figure 4). Viability of the cells exposed to high concentration of HAs did not significantly change in comparison to the intact cells.

Taking into account the experimental data on antioxidant and cell protective activity of HAs, these effects could be attributed to three factors:

1. High antioxidant activity that depends on concentration of phenolic components;
2. The ability of HAs to permeate a cell membrane and, possibly, interact with intracellular protective systems;
3. Low cytotoxicity even in very high concentration.

These results are in good agreement with the published data on some synthetic purified phenolic compounds [49].

### 3.3. Ontology-Based Model of the Antioxidant and Cytoprotective Activity

**Figure 5:**
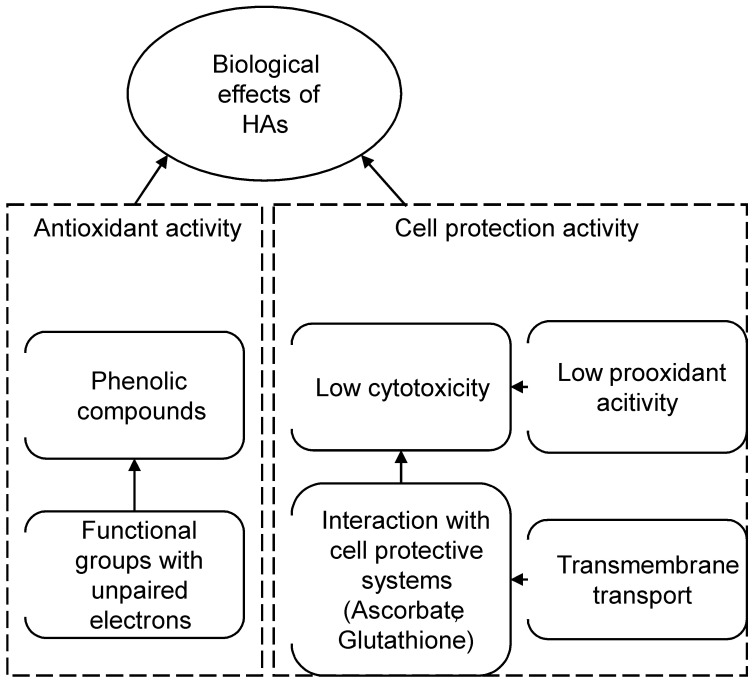
A hypothetical ontology-based model of biological activity of HAs.

Owing extremely high chemical complexity of HAs accompanied by diverse biological activity, HAs are among the most difficult natural substances to model their interaction with cellular metabolic pathways. The majority of common modelling frameworks, including quantum chemistry, chemical kinetics, and machine learning fails when it comes to simulating such a complex nonlinear system. Recently, a new ontology-based approach to describe heterogeneous biological processes has been introduced [39]. This type of model features inherent properties to colligate entities, which could represent either elementary or composite processes. One of the possible model describing biological activity of the HAs is given in Figure 5.

The model includes entities with different levels of complexity. The basic entities such as “phenolic compounds”, “transmembrane transport” etc. in fact are not elementary and could be refined further, but it requires detailed description and models of all processes along with their components and interactions. Moreover, excessive complexity does not guarantee better predictions and accuracy. The convenient feature of ontology-based models is their flexibility in decomposition. In theory, each entity can be itemized to the finest details. In practice, however, models should be as simple as possible provided they have desirable accuracy and precision.

All processes comprising the model can be approximated with essentially any mathematical tools at different levels of theory. For example, reducing ROS by functional groups could be simulated using a quantum mechanics/molecular mechanics (QM/MM) approach, whereas metabolic pathways can be described as a system of ordinary differential equations. Universal approximators such as ANNs and other machine learning frameworks might be very effective in some cases.

## 4. Discussion

Remarkable antioxidant and cell protective activity of HAs makes them a promising natural source of new pharmaceutical substances featuring a wide range of biological effects. Unfortunately, the exact biochemical mechanisms of these effects are remained unknown. Owing complexity of chemical structure, HAs cannot be modeled and simulated using common frameworks and software tools. Molecular docking and other standard modeling techniques are not applicable to HAs simply because we still do not know their chemical composition and structure. Numerical methods of quantum chemistry are also out of consideration for the same reason.

One of the possible approaches to overcome this obstacle is to decompose the intricate structure down to elementary entities that can be simulated using well-developed frameworks. Recently, ontology-based models of biological systems have demonstrated remarkable success solving problems of extremely high complexity. We believe that the same approach can be effectively used to tackle the complex biological processes involving HAs. The main drawback of the proposed model is lack of detailed descriptions and models of underlying biochemical processes, but these are not always necessary. 

We suggested an ontology-based model of antioxidant and cytoprotective activity of HAs based on experimental data and numerical models. This model establishes the way to further research on biological effects of HAs and provides a useful tool for numerical simulation of these effects.

## 5. Conclusions

Ontology-based models are gaining increasingly more attention in many areas of biomedical research because they provide a unique tool to describe very complicated structure–activity relationships at different levels of detail. Once some basic entities of the ontology model have been identified and modeled, these entities can be used as parts to construct another models and ontologies. This type of model can integrate virtually all known mathematical approaches to describe biological systems. Unfortunately, detailed simulation of metabolic pathways and biochemical reactions related to neurological or cardiovascular disorders, for example, are still beyond of the practical consideration. This issue can be resolved through studying, modeling and simulating of low level metabolical pathways as a structural foundation of ontological models. Our experimental findings confirmed that this approach does not contradict the previously published data. Moreover, we can also plan new experimental research purely based on numerical studies and numerically simulated prognosis. Although creating the ontology of the antioxidant activity of the HAs is at very early stage, the proposed approach appears quite promising and deserves further investigation.

## Figures and Tables

**Table 1:** Short description of the peat samples.

Peat Type	Sample Name	Sampling Depth (cm)	Degree of Decay (%)
Raised bog sphagnum	Peat 1	20–70	5–10
Raised bog pine-cotton-grass	Peat 2	10–50	30–35
Raised bog magellanicum	Peat 3	20–70	10–15
Raised bog fuscum	Peat 4	20–70	5–10
Low-mire woody	Peat 5	10–50	25–30
Low-mire grass-moss	Peat 6	200–250	35–50
Low-mire grass	Peat 7	230–270	40–45
Low-mire woody peat	Peat 8	50–100	30–35
Mesotrophic carex peat	Peat 9	150–200	40–45

**Table 2:** Antioxidant activity of the HAs, IC_50_ μg/mL.

Parameter	Min	Max
DPPH	5.2	20.8
ABTS	10.6	28.5
Fe chelating	26.9	100.0
OH	240.0	2590.0
Superoxide	3.9	38.1

**Table 3:** Linear correlations between antioxidant activity and concentration of hydroxyl groups (OH).

Parameter	r	R^2^	*p* Value
DPPH	−0.27	0.01	0.279
ABTS	0.30	0.03	0.222
Fe chelating	0.24	0.01	0.343
OH	−0.14	0.04	0.588
Superoxide	−0.03	0.06	0.903

**Table 4:** Linear correlations between antioxidant activity and concentration of carboxyl groups (COOH).

Parameter	r	R^2^	*p* Value
DPPH	0.07	0.05	0.781
ABTS	−0.20	0.01	0.451
Fe chelating	−0.18	0.02	0.464
OH	0.04	0.06	0.980
Superoxide	−0.03	0.06	0.887

**Table 5:** Linear correlations between antioxidant activity and the number of paramagnetic centers.

Parameter	r	R^2^	*p* Value
DPPH	−0.6	0.3	0.008 *
ABTS	0.07	0.05	0.768
Fe chelating	0.13	0.04	0.600
OH	−0.17	0.02	0.484
Superoxide	−0.26	0.01	0.291

Variables in the Table 3, Table 4, Table 5 and Table 6 are: r—coefficient of linear correlation; R^2^—the adjusted coefficient of determination; *p* value—the achieved confidence level, *—statistically significant values (*p* < 0.05).

**Table 6:** Linear correlations between measured values and those predicted with the PARAFAC model antioxidant activity.

Parameter	r	R^2^	*p* Value
DPPH	0.31	0.02	0.459
ABTS	0.64	0.33	0.02 *
Fe chelating	0.70	0.42	0.007 *
OH	0.57	0.24	0.049 *
Superoxide	0.73	0.48	0.002 *

r—coefficient of linear correlation, R^2^—the adjusted coefficient of determination, *p* value—the achieved confidence level, *—statistically significant values.

## Data Availability

Not applicable.

## Supplementary Materials

The following supporting information can be downloaded at: https://www.mdpi.com/article/10.3390/polym14163293/s1, Figure S1: Excitation–emission spectra of the studied peat samples; Table S1: Structural characteristics of humic acid samples used in this study: elemental composition, acidic functional groups, absorption spectroscopy and molecular weight distribution; Table S2: Structural characteristics of humic acid samples used in this study: ^13^C-NMR and FTIR spectroscopy; Table S3: Cytotoxicity analysis and antioxidant activity of humic acid samples used in this study.
