# Supplementary material for: Quantitative Structure-Activity Relationship, Ontology-Based Model of the Antioxidant and Cell Protective Activity of Peat Humic Acids
Source: Polymers (Basel). 2022 Aug 12;14(16):3293. doi: 10.3390/polym14163293 (PMC9412878; doi:10.3390/polym14163293)
Supplement: Supplementary file 1 [file polymers-14-03293-s001.zip › polymers-1775307-supplementary.pdf]

Figure S1. Excitation-emission spectra of the peat samples.

Sample | Alkaline extraction | Phosphate extraction

1

2

3

4

5

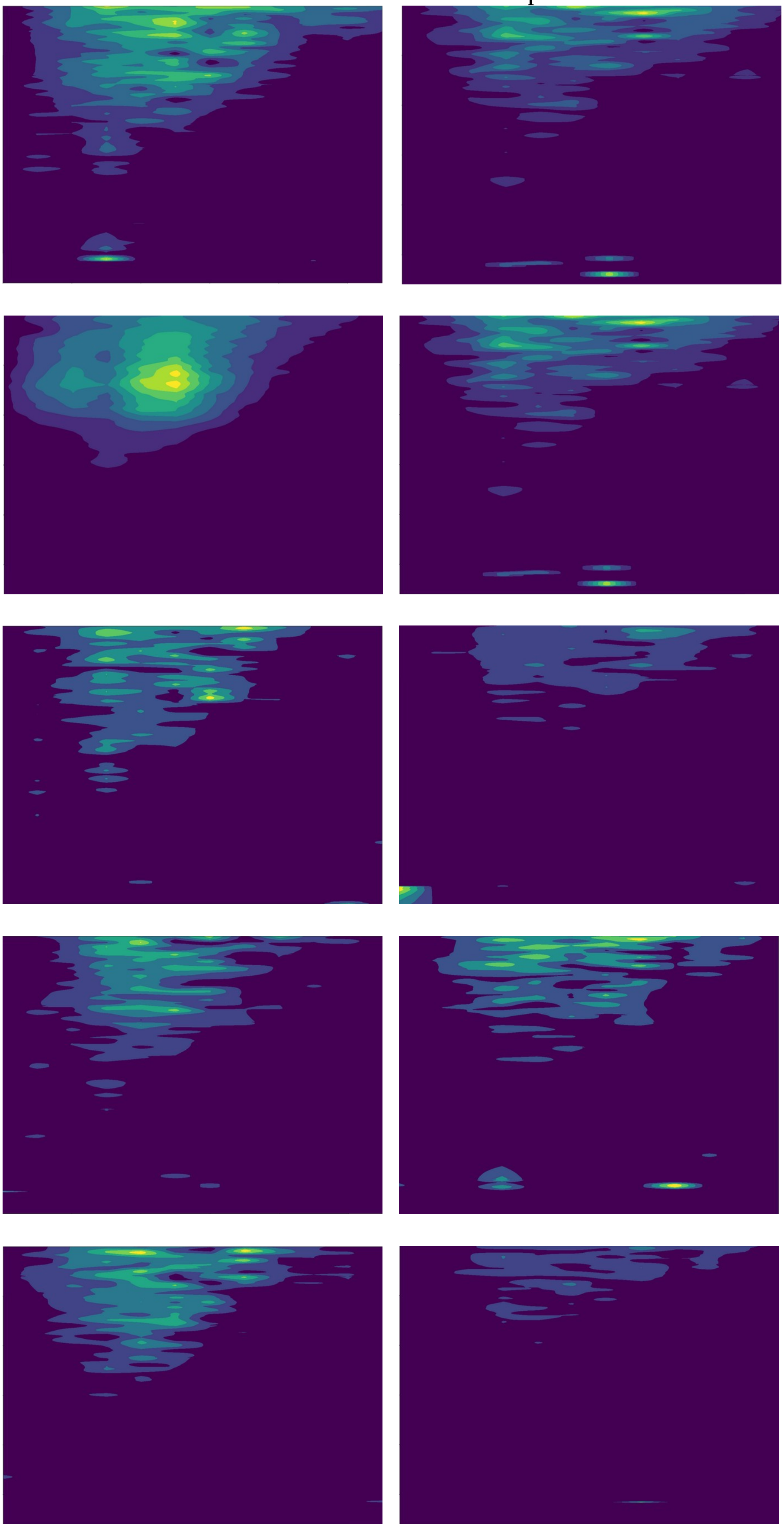

Table S1. Structural characteristics of humic acid samples used in this study: elemental composition, acidic functional groups, absorption spectroscopy and molecular weight distribution.

Sample	Elemental composition expressed as atomic percent and of H/C versus O/C						Acidic functional groups determined by chemical titration (mmol/g)			Absorption spectroscopy data			HPLC			
	**C**	**H**	**N**	**O**	**H/C**	**O/C**	**Total Acidity**	**COOH**	**OH phenolic**	**A 465**	**A 665**	**4:6**	**Mn, Да**	**Mw, Да**	**P**	**Mp, Да**
HAalk-1	35.73±1.07	47.65±1.43	2.25±0.07	14.37±0.43	1.35±0.05	0.40±0.01	5.20±0.03	2.48±0.01	2.72±0.06	0.12	0.04	2.85	7013.6	34568.6	4.9	15570.8
HAalk-2	35.95±1.08	46.16±1.32	1.62±0.04	16.27±0.48	1.30±0.04	0.45±0.01	5.76±0.01	2.84±0.04	2.92±0.02	0.13	0.04	2.91	5533.2	18968.1	3.4	10244.7
HAalk-3	35.73±1.07	46.78±1.40	2.22±0.06	15.27±0.45	1.32±0.04	0.43±0.01	5.67±0.03	2.44±0.01	2.24±0.06	0.15	0.05	3.17	7251.5	27757.5	3.8	14187.5
HAalk-4	35.76±1.07	47.32±1.42	2.00±0.05	14.92±0.44	1.34±0.04	0.42±0.01	6.14±0.03	2.60±0.02	3.54±0.06	0.13	0.04	2.91	8063.7	31423.7	3.9	15918.3
HAalk-5	36.46±1.09	44.77±1.34	2.09±0.05	16.68±0.51	1.24±0.03	0.46±0.01	5.86±0.02	2.76±0.01	3.10±0.03	0.12	0.04	2.90	5838.8	24430.0	4.2	11943.3
HAalk-6	37.17±1.11	43.26±1.29	2.07±0.05	17.50±0.52	1.17±0.03	0.47±0.01	5.88±0.01	2.78±0.01	3.10±0.01	0.15	0.04	3.41	5574.9	22073.9	4.0	11093.3
HAalk-7	37.44±1.12	43.70±1.31	2.31±0.08	16.55±0.49	1.18±0.03	0.44±0.01	6.72±0.03	2.62±0.01	4.10±0.06	0.18	0.06	3.25	5091.3	20830.8	4.1	10298.4
HAalk-8	36.12±1.08	44.83±1.34	2.26±0.07	16.79±0.50	1.25±0.03	0.46±0.01	5.69±0.02	2.57±0.01	3.12±0.03	0.13	0.04	3.18	4419.8	17638.4	4.0	8829.4
HAalk-9	37.16±1.11	44.09±1.32	1.84±0.04	16.91±0.51	1.20±0.03	0.45±0.01	5.70±0.01	2.59±0.03	3.12±0.01	0.14	0.04	3.13	4984.5	19970.0	4.0	9977.0
HApyr-1	35.05±1.05	44.60±1.33	2.75±0.09	17.60±0.53	1.28±0.03	0.50±0.01	5.72±0.02	2.37±0.01	3.35±0.03	0.06	0.04	1.50	7737.8	39698.0	5.1	17526.4
HApyr-2	38.57±1.16	42.46±1.27	1.40±0.04	17.57±0.53	1.11±0.03	0.45±0.01	6.76±0.02	2.65±0.01	4.11±0.04	0.12	0.06	2.04	6110.2	22783.9	3.7	11798.9
HApyr-3	38.44±1.15	42.47±1.27	2.20±0.06	16.89±0.50	1.11±0.03	0.43±0.01	6.05±0.02	2.75±0.01	3.30±0.03	0.10	0.04	2.15	4871.2	18755.3	3.9	9558.3
HApyr-4	36.87±1.11	42.17±1.25	2.09±0.05	18.87±0.57	1.15±0.03	0.51±0.01	6.07±0.02	2.46±0.01	3.61±0.03	0.06	0.04	1.53	6753.9	25782.1	3.8	13195.8
HApyr-5	40.11±1.20	41.89±1.25	2.06±0.05	15.94±0.46	1.05±0.03	0.40±0.01	5.95±0.03	2.80±0.01	3.15±0.05	0.08	0.05	1.68	5884.7	21203.3	3.6	11770.3
HApyr-6	38.18±1.14	41.50±1.24	2.22±0.06	18.10±0.55	1.10±0.03	0.47±0.01	6.16±0.02	2.63±0.01	3.52±0.03	0.06	0.05	1.34	5450.1	20906.6	3.8	10674.4
HApyr-7	40.26±1.20	40.08±1.20	2.27±0.07	17.39±0.52	1.00±0.02	0.43±0.01	7.52±0.03	2.68±0.01	4.84±0.06	0.06	0.04	1.48	4687.4	16707.7	3.6	8849.6
HApyr-8	39.65±1.19	40.65±1.22	1.99±0.05	17.71±0.53	1.03±0.03	0.44±0.01	6.52±0.02	2.99±0.01	3.53±0.04	0.08	0.05	1.68	4611.7	17233.9	3.7	8915.0
HApyr-9	38.99±1.17	41.78±1.25	1.97±0.05	17.26±0.51	1.08±0.03	0.44±0.01	6.18±0.01	3.08±0.01	3.10±0.02	0.07	0.05	1.38	4313.2	17508.5	4.1	8690.1

Table S2. Structural characteristics of humic acid samples used in this study: ^{13}C-NMR and FTIR spectroscopy.

Sample	Content of Carbon in Structural Fragments Determined by ^{13}C-NMR Spectroscopy as Integral Intensity (%)[1]									Fourier transform Infrared (FTIR) spectroscopy[2]										
	$C_{C=O}$	C_{COO}	C_{arO}	C_{ar}	C_{OCO}	C_{CHnO}	C_{CHn}	$\Sigma C_{ar}/\Sigma C_{alk}$	ΣC_{carb}	3400/1610	3400/2920	1720/1610	1720/2920	1225/1610	1225/2920	1035/1610	1035/2920	1035/1720	2920/1610	1610/2920
HAalk-1	2.4	12.8	7.8	19.3	7.7	24.3	25.6	0.47	32	0.62	0.81	0.89	1.15	0.85	1.09	0.79	1.02	0.89	0.77	1.29
HAalk-2	5.8	17	9.2	21.7	7	12.8	26.5	0.67	19.8	0.69	0.82	1.08	1.27	1.00	1.18	0.78	0.92	0.72	0.85	1.18
HAalk-3	9.6	13.4	7.9	20.8	6	19.3	22.9	0.6	25.3	0.75	1.01	0.88	1.14	0.82	1.06	0.76	0.98	0.86	0.77	1.30
HAalk-4	6.2	16.8	5.4	15.6	7.4	22	26.6	0.38	29.4	0.83	0.92	0.96	1.18	0.90	1.00	0.84	0.93	0.88	0.90	1.11
HAalk-5	4.2	16	7.4	25.3	6.6	20.5	19.9	0.7	27.1	0.71	0.99	0.95	1.32	0.97	1.35	0.85	1.19	0.90	0.72	1.39
HAalk-6	4	14.2	10	24.7	9.5	20.9	16.7	0.74	30.4	0.74	1.02	0.96	1.34	1.01	1.39	0.91	1.26	0.95	0.72	1.39
HAalk-7	5.7	13.9	12.2	27.3	7.8	20.4	12.7	0.97	28.2	0.92	1.16	0.95	1.20	0.95	1.19	1.01	1.23	1.07	0.79	1.26
HAalk-8	8.6	15.6	5.4	25	3.3	17.2	24.9	0.67	20.5	0.74	0.96	0.98	1.13	1.01	1.16	0.94	1.09	0.96	0.87	1.16
HAalk-9	6.7	14.7	11.1	22.7	9.8	15.7	19.3	0.75	25.5	0.73	0.87	0.98	1.17	0.98	1.18	0.85	1.13	0.96	0.83	1.20
HApyr-1	4.8	12.2	11.1	22.3	7.8	22.7	19	0.67	30.5	0.79	1.10	0.82	1.14	0.89	1.24	1.12	1.57	1.38	0.72	1.40
HApyr-2	3.3	13.1	9.6	26.8	4.4	24.2	18.7	0.77	28.6	0.96	1.19	0.96	1.20	0.89	1.12	0.81	1.02	0.85	0.80	1.23
HApyr-3	3.3	14.5	8.9	21.7	7.6	17.7	26.2	0.59	25.3	0.78	1.07	0.94	1.33	0.88	1.24	0.81	1.14	0.86	0.71	1.42
HApyr-4	6.9	18.8	6	22.5	6.6	12.3	26.9	0.62	18.9	0.84	1.06	0.94	1.06	0.90	1.14	0.93	1.17	0.99	0.79	1.26
HApyr-5	5.9	17.8	7.3	26.4	5.8	18.7	18	0.79	24.5	0.73	1.14	0.91	1.42	0.94	1.47	0.85	1.33	0.94	0.64	1.56
HApyr-6	4.5	13.4	12.4	27.5	7	21.4	13.9	0.94	28.4	0.83	1.29	0.86	1.33	0.84	1.30	1.06	1.64	1.22	0.64	1.56
HApyr-7	4	15.2	11.6	30.8	7.7	18.6	12.2	1.1	26.3	1.15	1.76	0.88	1.34	0.81	1.24	0.81	1.28	0.92	0.65	1.53
HApyr-8	4.2	13.7	10.4	27.9	5.4	16.5	21.8	0.88	21.9	0.83	1.14	0.91	1.41	0.93	1.44	0.76	1.17	0.83	0.65	1.55
HApyr-9	6.8	13.3	9.6	23.2	7.9	13	26.2	0.7	20.9	0.74	1.07	0.98	1.41	0.99	1.42	0.94	1.23	0.87	0.70	1.44

Note:

[1]Content of carbon in structural fragments was determined by ^{13}C NMR spectroscopy as integral intensity (%) of the following spectral regions (ppm): 220–187 ($C_{C=O}$). 187–165 (C_{COO}). 165–145 (C_{ArO}). 145–108 (C_{Ar}). 108–90 (C_{OCO}). 90–48 (C_{CHnO}). 48–5 (C_{CHn}).

[2]Fourier transform Infrared (FTIR) spectroscopy: 3400/1610 (phenol groups). 3400/2920 (aliphatic hydroxyls). 1720/1610 (aromatic oxo groups). 1720/2920 (aliphatic oxo groups). 1225/1610 (aromatic esters). 1225/2920 (aliphatic esters). 1035/1610 (aromatic ethers). 1035/2920 (aliphatic ethers). 1035/1720 (carbohydrates). 2920/1610 (aliphatic). 1610/2920 (aromatic)

Table S3. Cytotoxicity analysis and antioxidant activity of humic acid samples used in this study.

Sample	Viability of 3T3-L1 normal fibroblast cells %	Antioxidant activity						
		$ABTS^{\bullet +}$	DPPH	PMC	Voltammetry	$O_2^{-}\bullet$	Fe chelating	HO•
		µmol TE mg^{-1}	% Inhibition	(spin/g × 10^{-16})	(µmol/L × min^{-1})	IC_{50}		
						mcg/ml		mg/ml
HAalk-1	76.5±4.8	4.06±0.08	89.50±0.02	5.47±0.03	0.24±0.03	40.36±2.40	115.00±0.26	1.77±0.10
HAalk-2	72.2±2.3	1.31±0.05	91.10±0.02	6.60±0.03	0.37±0.03	25.31±0.90	115.95±4.28	1.09±0.06
HAalk-3	74.8±3.5	3.67±0.13	91.60±0.02	7.57±0.04	0.56±0.02	23.90±0.57	99.90±1.09	0.85±0.07
HAalk-4	73.6±3.8	0.80±0.27	92.80±0.02	7.80±0.04	0.61±0.03	36.21±2.06	74.41±0.76	0.61±0.04
HAalk-5	82.3±0.6	4.15±0.11	91.00±0.02	5.91±0.03	0.40±0.03	29.52±1.54	98.31±2.97	2.16±0.08
HAalk-6	81.4±7.1	1.34±0.06	93.10±0.02	7.93±0.04	0.58±0.04	20.99±1.24	106.60±1.16	1.38±0.13
HAalk-7	95.7±2.7	1.04±0.10	94.60±0.01	9.60±0.05	0.70±0.01	15.25±0.43	64.70±0.60	0.59±0.02
HAalk-8	81.0±4.4	0.95±0.05	84.30±0.02	5.42±0.03	0.30±0.01	36.00±1.07	96.54±2.46	1.61±0.14
HAalk-9	87.9±4.7	2.17±0.10	79.20±0.03	4.49±0.02	0.22±0.01	42.30±1.96	96.03±3.78	1.99±0.12
HApyr-1	86.4±3.6	1.70±0.13	87.80±0.02	5.05±0.03	0.23±0.02	57.36±0.59	81.20±2.26	2.02±0.08
HApyr-2	88.3±4.5	3.00±0.11	92.20±0.01	7.06±0.04	0.58±0.03	27.60±1.60	63.22±5.87	0.83±0.04
HApyr-3	87.2±4.6	1.24±0.41	91.10±0.02	5.55±0.03	0.35±0.02	23.71±0.74	84.25±1.79	0.83±0.04
HApyr-4	79.6±1.9	0.91±0.11	93.60±0.01	8.69±0.05	0.79±0.04	21.17±0.77	67.21±1.92	0.60±0.04
HApyr-5	79.8±3.8	0.51±0.07	92.00±0.02	6.78±0.04	0.53±0.01	32.96±0.81	87.07±0.61	1.29±0.08
HApyr-6	94.1±2.4	1.85±0.05	94.60±0.01	16.30±0.07	0.69±0.04	20.64±1.37	81.07±5.10	0.37±0.02
HApyr-7	82.3±3.3	1.19±0.08	94.80±0.01	14.40±0.06	0.91±0.03	19.16±1.39	58.18±1.91	0.33±0.02
HApyr-8	92.7±4.6	1.71±0.24	79.60±0.03	5.74±0.03	0.31±0.03	34.89±0.77	81.15±1.38	1.53±0.04
HApyr-9	81.7±5.2	2.01±0.04	92.10±0.01	6.90±0.04	0.41±0.03	31.32±0.89	97.32±0.72	0.86±0.02
